# Adaptation to Experimental Jet-Lag in R6/2 Mice despite Circadian Dysrhythmia

**DOI:** 10.1371/journal.pone.0055036

**Published:** 2013-02-04

**Authors:** Nigel I. Wood, Catherine J. McAllister, Marc Cuesta, Juliet Aungier, Eloise Fraenkel, A. Jennifer Morton

**Affiliations:** Department of Physiology, Development and Neuroscience, University of Cambridge, Cambridge, United Kingdom; Vanderbilt University, United States of America

## Abstract

The R6/2 transgenic mouse model of Huntington’s disease (HD) shows a disintegration of circadian rhythms that can be delayed by pharmacological and non-pharmacological means. Since the molecular machinery underlying the circadian clocks is intact, albeit progressively dysfunctional, we wondered if light phase shifts could modulate the deterioration in daily rhythms in R6/2 mice. Mice were subjected to four x 4 hour advances in light onset. R6/2 mice adapted to phase advances, although angles of entrainment increased with age. A second cohort was subjected to a jet-lag paradigm (6 hour delay or advance in light onset, then reversal after 2 weeks). R6/2 mice adapted to the original shift, but could not adjust accurately to the reversal. Interestingly, phase shifts ameliorated the circadian rhythm breakdown seen in R6/2 mice under normal LD conditions. Our previous finding that the circadian period (tau) of 16 week old R6/2 mice shortens to approximately 23 hours may explain how they adapt to phase advances and maintain regular circadian rhythms. We tested this using a 23 hour period light/dark cycle. R6/2 mice entrained to this cycle, but onsets of activity continued to advance, and circadian rhythms still disintegrated. Therefore, the beneficial effects of phase-shifting are not due solely to the light cycle being closer to the tau of the mice. Our data show that R6/2 mice can adapt to changes in the LD schedule, even beyond the age when their circadian rhythms would normally disintegrate. Nevertheless, they show abnormal responses to changes in light cycles. These might be caused by a shortened tau, impaired photic re-synchronization, impaired light detection and/or reduced masking by evening light. If similar abnormalities are present in HD patients, they may suffer exaggerated jet-lag. Since the underlying molecular clock mechanism remains intact, light may be a useful treatment for circadian dysfunction in HD.

## Introduction

It is now well established that sleep disruption and changes to circadian cycles of activity are important symptoms of neurodegenerative diseases such as Alzheimer’s disease [1], Parkinson’s disease [2], and Huntington’s disease (HD) [3]. We have shown previously that the sleep/wake dysfunction seen in HD patients is recapitulated in the R6/2 mouse, a transgenic mouse model of HD [3]. The R6/2 transgenic mouse was one of the first, and is the best characterized, model of HD. It expresses exon 1 of the HD gene, with an expanded CAG repeat [4]. The R6/2 mouse displays many features of the symptoms that are seen in human HD patients, including motor, cognitive, emotional and social impairments [5]–[7]. R6/2 mice also display a disintegration of their daily rhythms of rest and activity, and disruption to the temporal expression of both clock genes and clock-controlled genes *in vivo* [3], [8]–[9]. However, when the suprachiasmatic nuclei (SCN) are removed from dysrhythmic mice and cultured *in vitro*, the endogenous rhythm of clock gene expression is normal [8]. This suggests that the molecular machinery underlying circadian rhythms generated by the SCN is intact, and raises the possibility that the circadian dysfunction in R6/2 mice may respond to treatments aimed at activating the deficient pathways afferent to and/or efferent from the SCN. In support of this, we have demonstrated that the pharmacological imposition of sleep through administration of the sedative alprazolam can produce improvements in rest/activity cycles, and also in cognitive behavior [8]. In addition, improvements in daily rhythms were obtained through the imposition of a regime of time-restricted feeding [9]. Since the circadian rhythms of R6/2 mice can be improved through both pharmacological and non-pharmacological means, we were interested in determining whether or not R6/2 mice were capable of responding appropriately to a challenge to their circadian mechanisms invoked by alterations in the light/dark cycle. It is known that R6/2 mice have retinal degeneration [10], which may cause visual defects. Although it has not been studied, it is possible that these histological abnormalities extend to the intrinsically photosensitive retinal ganglion cells; thus retinal degeneration may contribute to the loss of circadian rhythmicity. We planned to subject the mice to repeated light phase-shifts of 4–6 hours, and monitor how well (and for how long) their daily activity rhythms adapted to the new time of light onset. However, there is a potential difficulty in using this approach, since we would, in effect, be inducing jet-lag in the mice. It is well known that the disruption to the circadian system that is a consequence of jet-lag can impact on cognitive performance and health [11]–[12], as a result of de-synchronization between Zeitgebers and the endogenous clockwork. It has also been found that inducing experimental jet-lag in mice has deleterious effects on age at death, and that advances in the light cycle have a more profound effect than delays [13]. Therefore, in addition to testing a set of serial advances in light onset, we tested the response of R6/2 mice to a second experimental jet-lag regime (a shift/reversal paradigm that comprised 6 hours phase-advance followed by 6 hours phase-delay, and vice versa). As well as providing information as to whether or not R6/2 mice could adapt to these changes in their light cycles, we anticipated that this study could also provide clues as to how well HD patients might cope with jet-lag.

## Methods

### Ethics Statement

All components of this study were carried out in accordance with the UK Animals (Scientific Procedures) Act, 1986, and with the approval of the University of Cambridge Licence Review Committee.

### Animals

Mice were taken from a colony of R6/2 transgenic mice [4] established in the Department of Pharmacology, University of Cambridge, and maintained by backcrossing onto CBA x C57BL6N F1 female mice. All R6/2 mice were hemizygotic. WT mice used were littermates of the R6/2 mice. Genotyping and CAG repeat length measurement were carried out by Laragen (Los Angeles, CA, USA) as previously described [14]. The 101 transgenic mice used in this study had a mean CAG repeat length of 254±1 (range 244–269).

Before the start of the experiments, mice were kept from weaning in home cages comprising single sex, single genotype groups of 10. All of the mice lived in an enhanced environment with increased amounts of bedding and nesting materials. Clean cages were provided twice weekly, with grade 8/10-corncob bedding, and finely shredded paper for nesting. The mice were maintained on a 12∶12 hour light/dark (LD) cycle, at a temperature of 21–23°C and a humidity of 55±10%. The mice had *ad libitum* access to water (using water bottles with elongated spouts) and dry laboratory food (RM3(E) rodent pellets, Special Diet Services, Witham, UK).

For the measurement of daily activity experiments, mice were singly housed in light- and sound-proof cabinets with a built-in light adjustment kit (Scanbur A/S, Denmark; light intensity 100 lux). Light at this intensity has been shown to be effective in maintaining circadian rhythms in C57Bl/6 mice [15]. A passive infra-red (PIR) sensor (model DS936, Bosch, Germany) was placed on each cage to monitor the activity of individual mice. We used PIRs because previous experience has shown that running wheels are not suitable for measuring activity in R6/2 mice. As the phenotype develops, the mice become ataxic [5]. Although they still move around the cage, they lose the strength and coordination (and perhaps the motivation) to use the running wheels. For this reason, PIRs are a better option for collecting activity data over the lifetime of the mice. The limitation of PIRs is that because they are much more sensitive than wheels, onsets and offsets of activity are less easy to determine, even by algorithms. We used ClockLab (Actimetrics, Wilmette, USA) to collect and analyze the data, and allowed the program’s algorithms to detect onsets, offsets, amplitudes and acrophases. ClockLab defines onset as 6 hours of inactivity followed by 6 hours of activity (and vice versa for offsets). As noted above, the sensitivity of the PIRs means that there are rarely periods of absolutely zero activity, but the template still fits the onsets and offsets. These look, to the eye, like reasonable positions. Therefore, the template must be fitting according to periods of relatively high and relatively low activity for each mouse. Although not perfect, this approach avoided potential bias from operators quantifying the data by hand. General activity data were double-plotted in actograms using 5 minute bins. Quantification of activity during light and dark phases was performed on 7 days of continuous data collected in WT and R6/2 mice. The onset of activity was estimated using a template-matching algorithm (ClockLab), with onset defined by the program as 6 hours of activity following 6 hours of inactivity (and vice versa for offsets). To avoid bias, we used the ClockLab program to identify onset times for each day throughout the experiment. We then interpolated onsets to make solid lines. χ^2^ periodogram analysis was performed and the amplitude of the periodogram was used to assess the robustness of the rhythm. Where the χ^2^ periodogram gave a non-significant value, the mouse was excluded from further analysis of activity onset. Acrophase was also estimated using the template-fitting algorithm in ClockLab. The day/night activity ratio reflects the quantity of activity occurring during the day (rest period in mice) as a fraction of quantity of activity occurring during the night (active period in mice). Typically in nocturnal species such as mice, it is the case that the smaller the ratio, the stronger the rest-activity rhythm. The phase angle of photic entrainment was calculated as the time of onset of activity for each mouse, relative to the time of lights off. For the phase-shift experiment we calculated the time taken for mice to achieve 50% of each phase-shift (PS_50_) [16]. True phase angles are best measured by releasing mice into DD conditions, then projecting a regression line to activity onsets in the dark up to the last day in the LD cycle. A measure of the time (distance) between lights off and the projected onset then gives the phase angle. However, although not an ideal measure, phase angles measured under LD give an estimation of when the circadian cycles are breaking down in R6/2 mice. For amplitude, acrophase, and light/dark ratio calculations, data were averaged across consecutive seven day periods for each mouse, giving a single data point for each week of age.

It is standard in circadian biology to use male animals only, since male activity is not influenced by the estrus cycle. However, HD affects both sexes equally. Furthermore, we were studying the effect of the HD mutation on circadian rhythms, and not circadian rhythms *per se*. We therefore thought it important to include mice of both sexes in our experiments.

Gaps in the data (seen in the actograms) were caused by either power cuts or mice chewing through the wires connecting the PIRs. There were four power cuts lasting 10 hours or more (see Table S1 for details). Mice in the shift/reversal experiment were tested in two balanced batches, so not all mice were affected by the power cuts.

### Normal Light/dark Conditions

Female WT (n = 11) and R6/2 (n = 23) mice were singly housed under normal light conditions (12∶12 LD, lights on 6am, off 6pm) from 5.5–17 weeks of age. Food and water were available *ad libitum*. The transgenic mice used in this component of the study had CAG repeat lengths of 261±1 (range 256–269).

### Serial Repeated Phase-advances

WT (n = 12, 6 female and 6 male) and R6/2 (n = 23, 12 female and 11 male) mice were singly housed under normal 12∶12 LD conditions (initially lights on 6am, off 6pm) from 6 weeks of age for 2 weeks (LD cycle 1). At 8 weeks of age, the light cycle was then advanced by 4 hours (i.e. lights on 2am, off 2pm; LD cycle 2). After a further 2 weeks under these conditions (at 10 weeks of age), the light cycle was again advanced by 4 hours (lights on 10pm, off 10am; LD cycle 3). This was repeated a total of 4 times, with the final shift occurring when the mice were 14 weeks of age. The transgenic mice used in this component of the study had CAG repeat lengths of 254±1 (range 244–269).

### Shift/reversal Paradigm

WT (n = 24, 12 female and 12 male) and R6/2 (n = 48, 24 female and 24 male) mice were singly housed under normal 12∶12 LD conditions (lights on 6am, off 6pm) from 12 weeks of age. After 3 weeks (when the mice were 15 weeks of age), 12 WT and 24 R6/2 mice were subjected to a 6 hour delay in the light cycle (lights on at 12 midday, off at 12 midnight). The remaining 12 WT and 24 R6/2 mice were subjected to a 6 hour advance in the light cycle (lights on at 12 midnight, off at 12 midday). All mice lived under this new lighting regime for 2 weeks (until they were 17 weeks of age), after which they were returned to normal LD conditions (lights on at 6am, off at 6pm). Thus, one group of the mice was subjected to a temporary phase-advance (analogous to travelling eastward), while the second group experienced a temporary phase-delay (analogous to a westward journey). The transgenic mice used in the phase-delay component of the study had CAG repeat lengths of 250±1 (range 244–256); mice in the phase-advance arm had CAG repeat lengths of 250±1 (range 245–255).

### 23 Hour Light/dark Cycle

We noticed during the previous experiments that the endogenous period of R6/2 mice appears to shorten, a finding that was confirmed by separate study [17]. Therefore, we kept a cohort of mice under a shortened light/dark cycle, to see if the R6/2 mice could maintain entrainment under conditions that conformed to their endogenous period. WT (n = 18, 9 female and 9 male) and R6/2 (n = 18, 9 female and 9 male) mice were singly housed under normal 12∶12 LD conditions (lights on 7am, off 7pm) for 2 weeks from the age of 9 weeks. Following this habituation period, the mice were switched to 23 hour light cycle (11.5∶11.5 LD) for the remainder of the lifespan of the R6/2 mice.

### Survival

Age of death was recorded for all R6/2 mice in the shift/reversal experiment. Mice were killed at end stage, i.e. if they were moribund, lacked a righting reflex, failed to rouse for their mash, or did not respond to gentle stimulation.

### Statistics

Group comparisons were made using Student’s t-test or ANOVA with repeated measures where appropriate. Differences between pairs of groups were evaluated using Bonferroni’s post-hoc test. Survival data were analyzed using a log-rank test. Statistical significance was set at p<0.05 except for determining circadian period length, where the significance level was pre-set by the ClockLab software (Χ^2^ periodogram) at p<0.001. Statistical analyses were performed using StatSoft Statistica 19.0 (StatSoft Inc., Tulsa, USA) or Prism 5 (GraphPad Software Inc., San Diego, USA).

## Results

### Daily Rhythms Under Normal LD Conditions Disintegrate in R6/2 Mice

**Figure 1:**
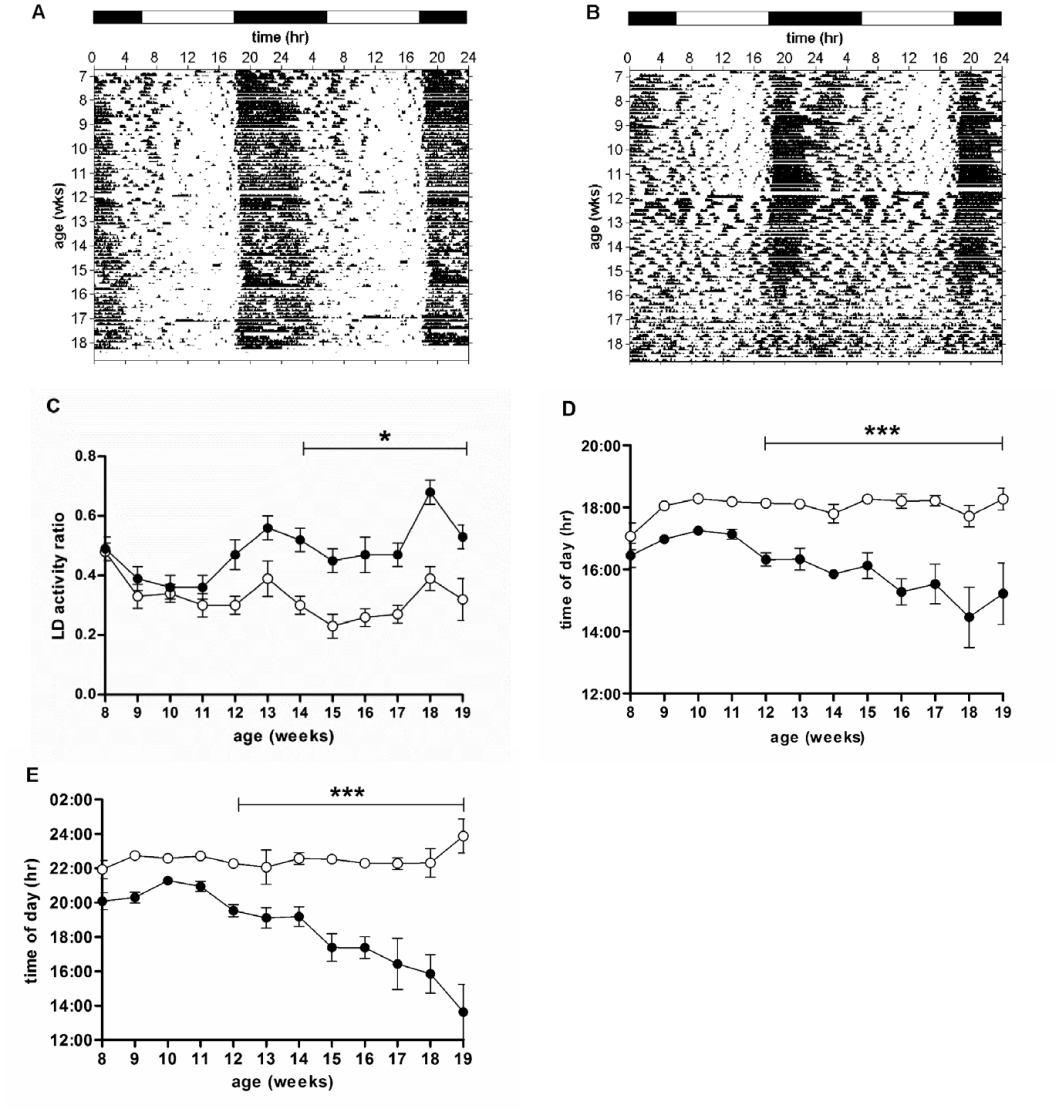
Disintegration of daily rhythm of activity in R6/2 mice. Double-plotted actograms from representative WT (A) and R6/2 (B) mice measured under LD conditions (12 h:12 h). Light/dark activity ratio (C), onset of activity (D), and acrophase (E) were averaged across 7 days. Open symbols are WT mice, filled symbols are R6/2 mice. Data are means ± SEM. *** = p<0.001.

**Figure 2:**
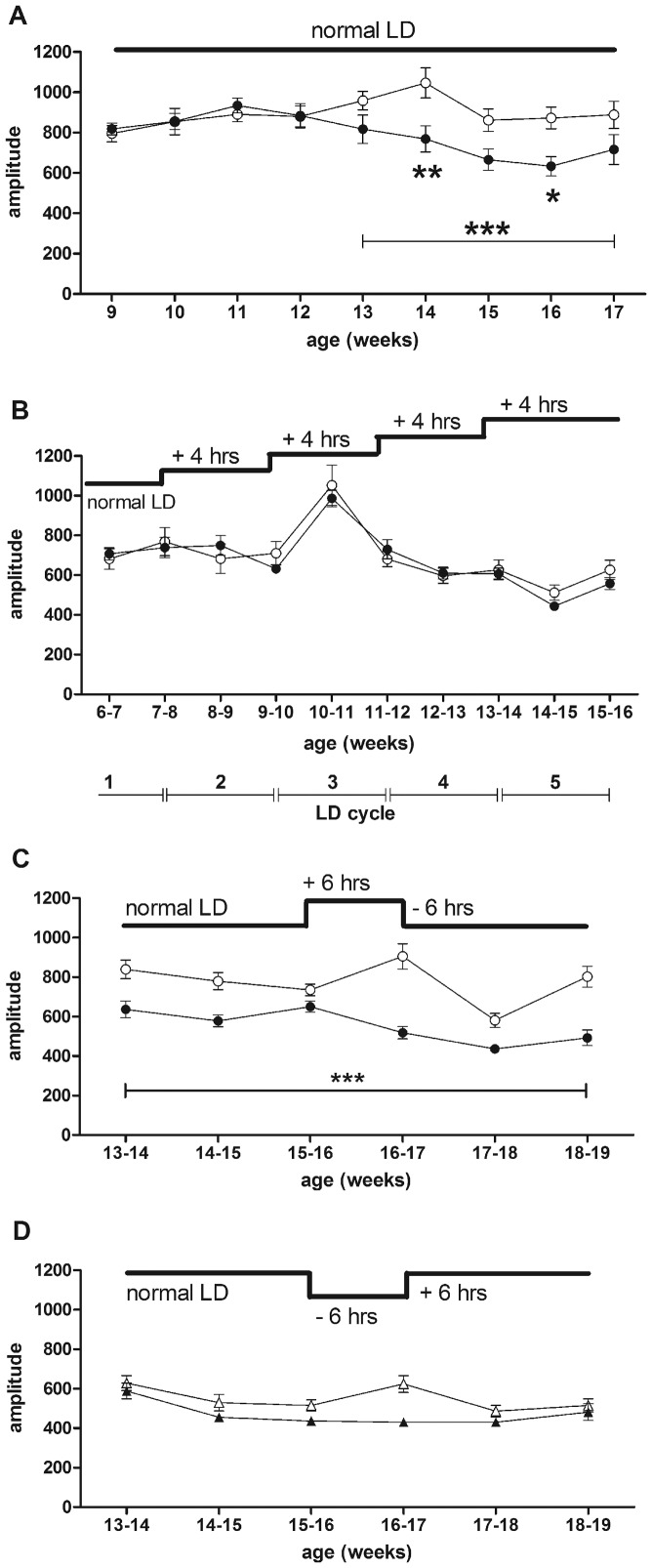
Amplitude of the daily activity cycle, averaged over every 7 day period. Results are shown from mice being kept under constant LD conditions (A), subjected to repeated phase shifts (B), and following jet-lag phase-advance (C) and phase-delay (D). WT mice are shown as open symbols. R6/2 mice are represented by filled symbols. Data are means ± SEM. * = p<0.05, ** = p<0.01, *** = p<0.001.

As previously shown, all WT mice showed a clear daily pattern of activity under LD conditions (12 h:12 h) from 7–19 weeks of age (Figure 1A, Figure S1). By contrast, R6/2 mice started to show a breakdown of daily activity rhythms by around 12 weeks of age (Figure 1B, Figure S1). We also calculated the amplitude of the daily activity cycle. This analysis revealed an age-related decrease in amplitude in R6/2 mice from 13 weeks of age (Figure 2A). We carried out a further analysis of the light/dark activity cycles, which showed that the light/dark activity ratio was greater in R6/2 mice from 14 weeks of age (p<0.05, Figure 1C), indicating a disruption to the daily cycle of activity. This conclusion was supported by analysis of onsets of activity, which showed from 12 weeks of age the onset of activity in R6/2 mice anticipated lights off by a steadily increasing amount (p<0.001, Figure 1D). Acrophase of activity in R6/2 mice showed a similar pattern, with acrophase occurring progressively earlier in R6/2 mice from 12 weeks of age (p<0.001, Figure 1E). These data suggest that there was an impairment of daily re-synchronization, or reduced masking in response to evening light.

### Daily Rhythms in R6/2 Mice are Maintained Under Repeated Phase-advances

**Figure 3:**
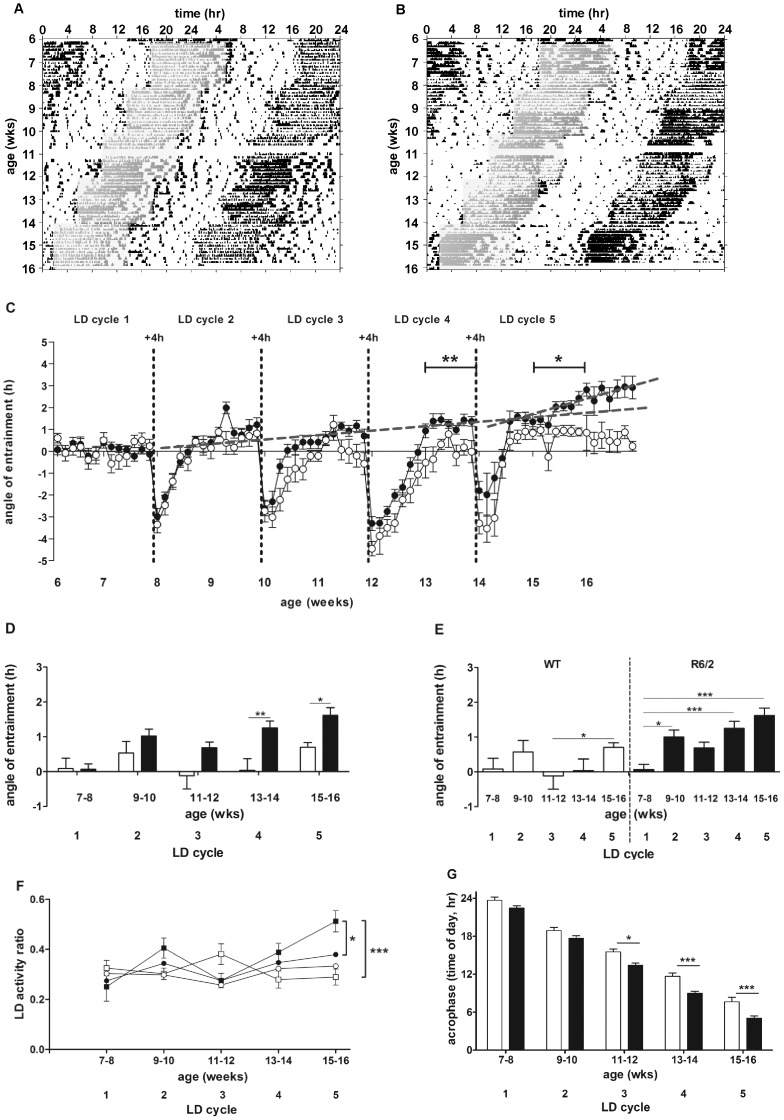
Double-plotted actograms from mice subjected to repeated 4 hour phase-advances (shaded blocks). Representative WT mice are shown in (A), and R6/2 mice in (B). Phase angles of entrainment throughout the study are shown in C. The dotted lines in C indicate linear regression in R6/2 mouse data from 8–14 weeks, and 14.5–17 weeks. Inter-genotype comparisons of angles of entrainment from the second week of each phase-advance are shown in D. E shows intra-genotype comparison of phase angles from the second week of each phase-advance. F shows the light/dark (LD) activity ratios under normal 12∶12 LD conditions (squares) and following repeated phase-advance (circles). G shows average acrophase for the second week following each phase-advance. In C-G, WT mice are shown as open symbols/columns, R6/2 mice are represented by filled symbols/columns. Data in C-G are means ± SEM. * = p<0.05, ** = p<0.01, *** = p<0.001.

Both WT (Figure 3A, Figure S2) and R6/2 (Figure 3B, Figure S3) mice adapted to repeated 4 hour phase-advances. We found no difference in amplitude between genotypes (Figure 2B). Thus, repeated phase-advances ameliorated the breakdown of daily activity rhythms that is typically seen in R6/2 mice under normal LD conditions.

Analysis of the phase angle of entrainment confirmed that both WT and R6/2 mice adapted to phase-advances (Figure 3C). Even at 15 weeks of age, R6/2 mice entrained to the phase-shift, although they were beyond the age at which the daily rhythm of activity typically becomes disrupted under constant LD conditions [3]. However, whereas the phase angle remained relatively constant over time in WT mice, it was significantly more positive in R6/2 mice (Figure 3E). There was a main effect of genotype (F_(1, 165)_ = 19.71, p<0.001), with R6/2 mice having angles of entrainment that were significantly more positive than those of WT littermates at 13–14 and 15–16 weeks of age (p<0.01 and p<0.05 respectively; Figure 3C, D). Relative to that observed in R6/2 mice at 7–8 weeks, angles in R6/2 mice were significantly more positive at 9–10 weeks (p<0.05), 13–14 weeks and 15–16 weeks (both p<0.001; Figure 3E). Analysis of LD activity ratios under normal 12∶12 LD conditions revealed a significant difference between WT and R6/2 mice by 15 weeks of age (p<0.001, Figure 3F). There was no difference between genotypes at the same age in mice that had been subjected to repeated phase-shifts (Figure 3F). Furthermore, subjecting R6/2 mice to phase-shifting significantly improved their LD ratios compared to R6/2 mice under normal LD conditions (p<0.05, Figure 3F). Because onsets of activity in R6/2 mice can become indistinct as the phenotype develops, we also measured acrophase as a second measure of the robustness of daily activity rhythms. At 7–8 weeks and 9–10 weeks, acrophase was not affected by genotype. However, acrophase in the R6/2 mice was significantly phase-advanced at 11–12 (p<0.05), 13–14 and 15–16 weeks of age (p<0.001) compared to WT mice (Figure 3G).

**Figure 4:**
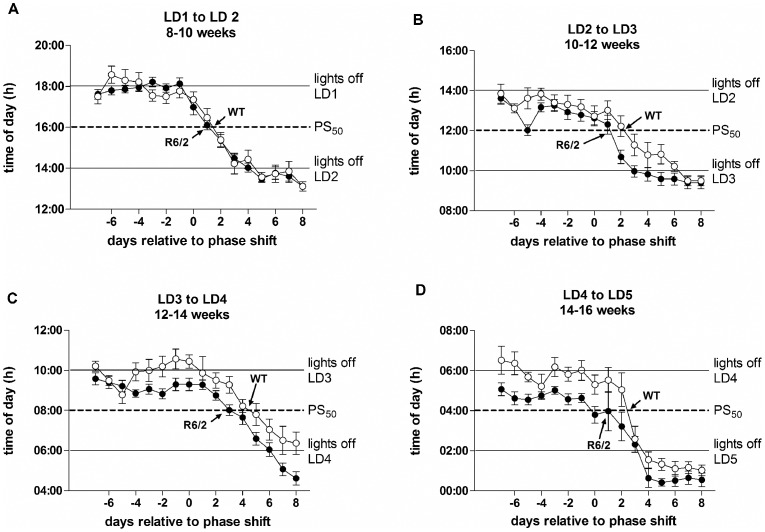
Average time of onset of activity of mice subjected to repeated phase-shifts, showing time to PS_50_ (the time at which half of the phase-shift was achieved). Light phase-shifts were conducted between 8–10 (A), 10–12 (B), 12–14 (C) and 14–15 (D) weeks of age. All values are means ± SEM. Open symbols are WT mice, filled symbols are R6/2 mice.

At the start of each 4 hour light phase-shift, we counted the number of days taken for the onset of activity of the mice to advance by 2 hours, i.e. the half-way stage. This point is designated PS_50_ [16]. For example, when the time of lights on shifted from 1800 to 1400, PS_50_ is at 1600. WT and R6/2 mice showed comparable PS_50_ times in shifting to the new light cycles (Figure 4, Table 1). There was a trend for R6/2 mice to entrain faster than WT mice that was already apparent by 10 weeks of age; but this difference was significant only at the shift from LD2 to LD3 (p<0.05; Figure 4B, Table 1). This apparent improvement in rate of entrainment in R6/2 mice may have been caused by an age-dependent increase in the angle of entrainment (Figure 3D, E). This echoes the results obtained from R6/2 mice held under constant light/dark conditions, which showed progressively earlier onsets of activity (Figure 1D). To take this into account, we re-calculated PS_50_ by zeroing the activity onset time to the onset of the final day under each LD cycle (Table 2). The results were similar to those obtained with raw data, although the difference between R6/2 and WT mice at the shift from LD2 to LD3 was more pronounced (p<0.01).

**Table 1:** Inter-genotype comparison of number of days taken to reach PS_50_ in the serial phase-advance experiment, analyzed using raw data.

	Number of days to PS_50_ (mean ± SEM)		
	WT	R6/2	P value
LD1 - LD2	2.2±0.2	1.9±0.2	p = n.s.
LD2 - LD3	3.3±0.7	1.8±0.2	p<0.05
LD3– LD4	5.3±0.7	3.9±0.4	p = n.s.
LD4 - LD5	2.5±0.4	1.5±0.4	p = n.s.

LD1-5 =  successive phase-shifts.

PS_50_ =  number of days for the onset of activity of the mice to advance by 2 hours during each 4 hour phase-shift.

**Table 2:** Inter-genotype comparison of number of days taken to reach PS_50_ in the serial phase-advance experiment, analyzed using zeroed data.

	Number of days to PS_50_ (mean ± SEM)		
	WT	R6/2	P value
LD1 - LD2	2.4±0.3	2.5±0.2	p = n.s.
LD2 - LD3	3.7±0.4	2.5±0.2	p<0.01
LD3– LD4	4.0±0.5	4.3±0.4	p = n.s.
LD4 - LD5	2.6±0.2	3.7±0.9	p = n.s.

LD1-5 =  successive phase-shifts.

PS_50_ =  number of days for the onset of activity of the mice to advance by 2 hours during each 4 hour phase-shift.

Onset times were zeroed to the final day before each phase-shift.

### R6/2 Mice can Adapt to Phase-shift and Reversal

**Figure 5:**
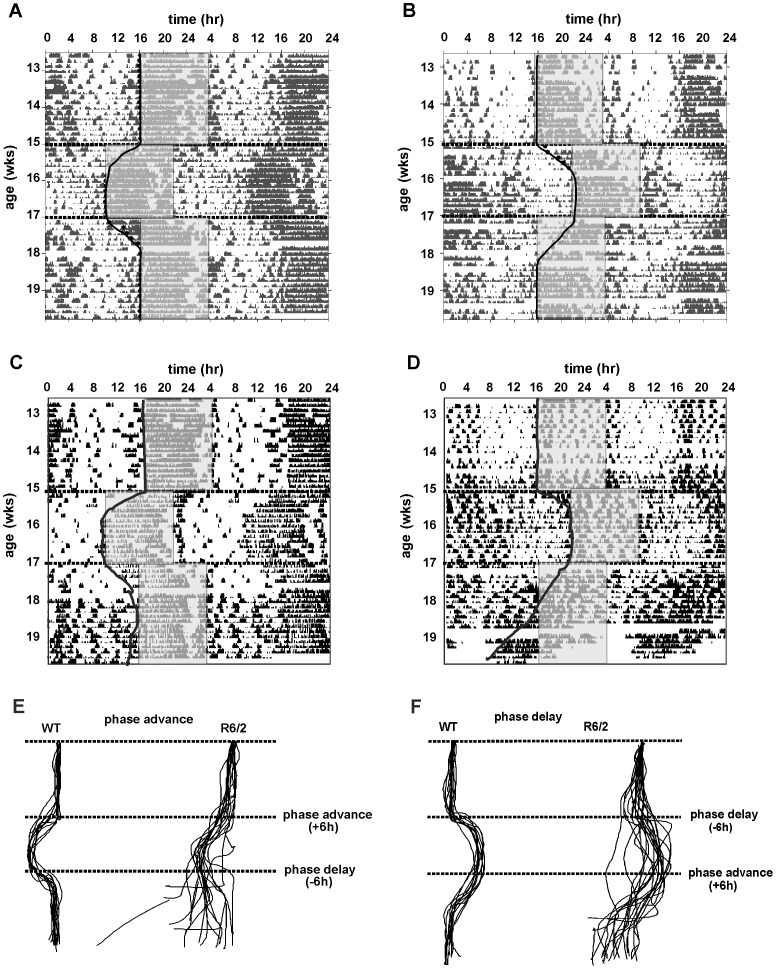
Representative double plotted actograms from mice undergoing phase-advance and phase-delay. WT (A, B) and R6/2 (C, D) mice were exposed to either phase-advance (A and C) or phase-delay (B and D). Shaded regions represent dark phase of LD cycle. Lines were drawn to plot onsets of activity for each mouse (as shown for representative mice in A-D). Lines from all mice are shown superimposed in E and F.

**Figure 6:**
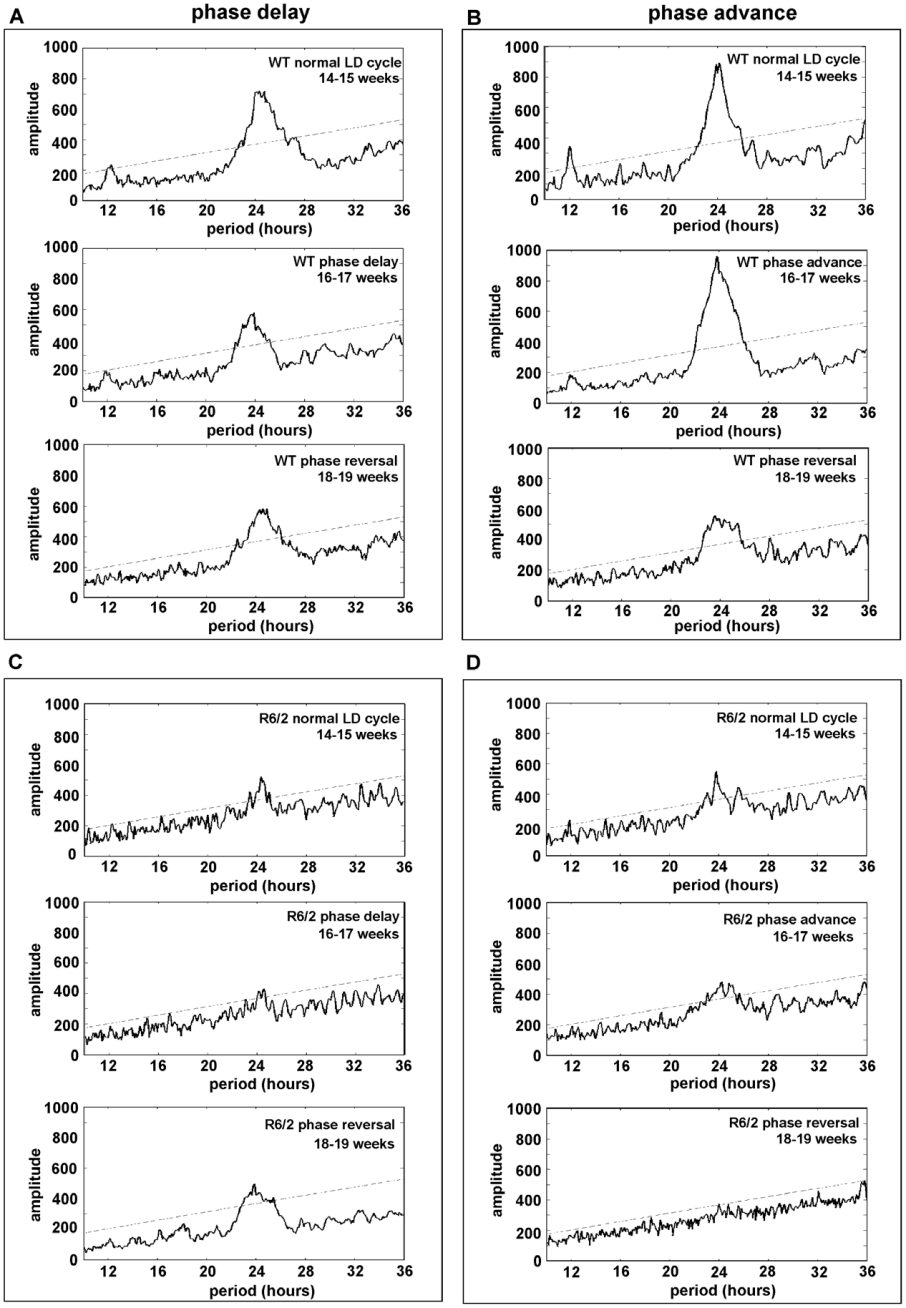
Periodograms from the second week of each phase-shift in the shift/reversal experiment from representative WT and R6/2 mice. Panel A shows a WT mouse undergoing phase-delay and reversal, Panel C an R6/2 mouse under the same conditions. Panel B shows a WT mouse undergoing phase-advance and reversal, Panel D an R6/2 mouse under the same conditions. The amplitude decreased with age in R6/2 mice, but was more strongly maintained under conditions of phase-delay with phase-advance as the reversal, than under phase-advance with phase-delay as the reversal. The dotted line in the periodograms represents significance at p<0.001.

**Figure 7:**
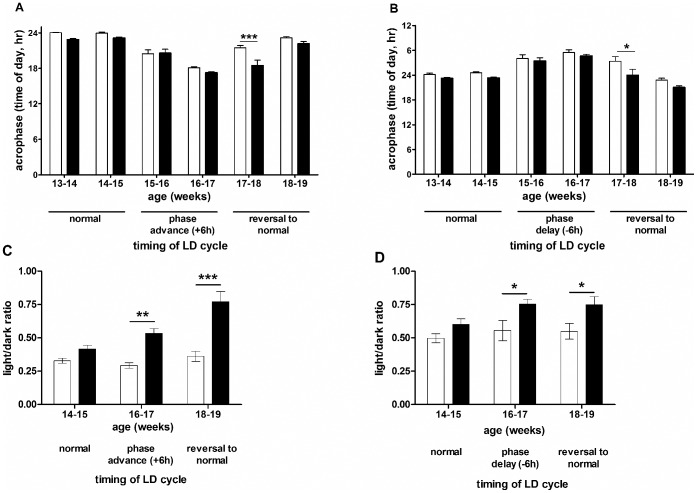
Acrophase and light/dark ratios in the shift/reversal experiment. Acrophase (A and B) and light/dark ratios (C and D) for WT (open columns) and R6/2 (filled columns) mice are shown following both phase-advance (A, C) and phase-delay (B, D). Data in A and B are means ± SEM of the acrophase in each week of the experiment. Data in C and D are the light/dark ratios from the second 7 days of each cycle. * = p<0.05, ** = p<0.01, *** = p<0.001.

WT and R6/2 mice shifted their onset of activity in response to both 6 hour phase-advance (Figure 5A, C, E, Figure S4) and phase-delay (Figure 5B, D, F, Figure S5), although R6/2 mice showed less precision in re-setting onset times at 15 weeks of age (Figure 5C, D, E, F). On return to a normal LD cycle, WT mice quickly re-adjusted to the new light onset time, irrespective of the direction of the shift (Figure 5A, B, E, F). By contrast, R6/2 mice were impaired in the re-adjustment (Figure 5C, D, E, F). Mice of both genotypes maintained significant activity rhythms in both the phase-delay (Figure 6A, C) and phase-advance (Figure 6B, D) conditions, although the daily rhythms became less distinct in R6/2 mice as the phenotype developed (Figure 6B, D). Analysis of amplitude revealed a significant genotype effect following phase advance (p<0.001, Figure 2C), but not after phase delay (p>0.05, Figure 2C). However, there was also a significant difference in amplitude between WT groups subjected to advance or delay, even before the phase shift (p<0.0001, Figure 2C). There was no difference between R6/2 mice subjected to either phase advance or delay (p>0.05, Figure 2C). Analysis of acrophase confirmed the presence of significant activity rhythms in R6/2 mice, and a delay (relative to WT mice) in re-entraining to the normal LD cycle following either phase-advance (p<0.001, Figure 7A) or phase-delay (p<0.05, Figure 7B). Calculation of LD ratios revealed an increase in ratios over time in R6/2 relative to WT mice (phase-advance: 16–17 weeks, p<0.01, 18–19 weeks, p<0.001, Figure 7C; phase-delay: 16–17 and 18–19 weeks, p<0.05, Figure 7D). This increase was present during the initial phase-shift period, suggesting that even though the R6/2 mice were able to re-entrain to a timing shift, the underlying dysfunction (breakdown in daily cycling) was still present.

**Figure 8:**
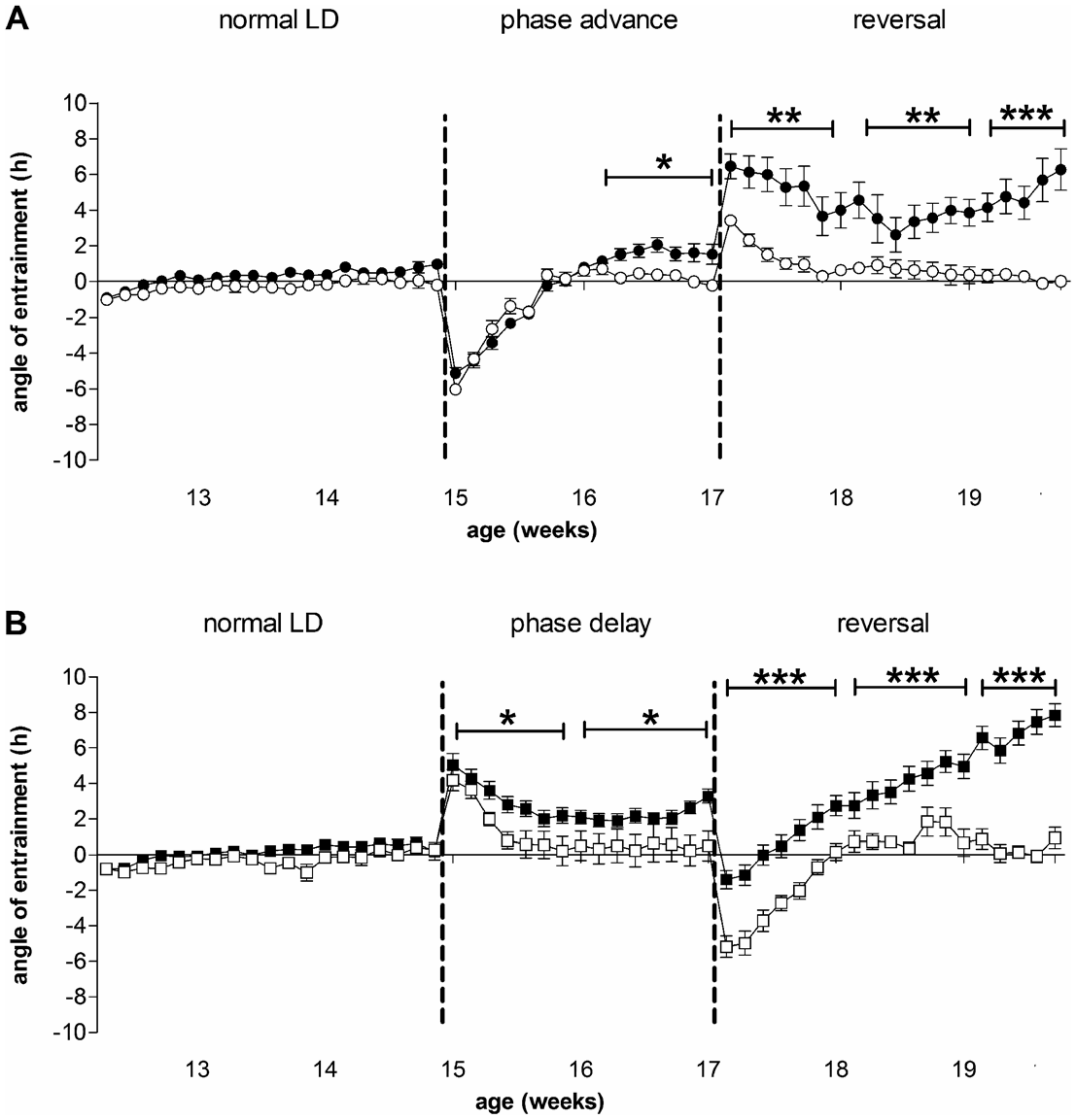
Phase angles of entrainment in the shift/reversal experiment. Mice were kept under normal LD conditions until 15 weeks of age, and were then subjected to two weeks of phase-advance followed by reversal to normal LD conditions (A), or to two weeks of phase-delay followed by reversal (B). Dotted lines indicate the start of each phase-shift. Open symbols are WT mice, filled symbols are R6/2 mice. Data are means ± SEM. * = p<0.05, ** = p<0.01, *** = p<0.001.

Although the R6/2 mice showed evidence of re-synchronization to both phase-shifts and reversals, they were impaired compared to WT mice. This was shown by the increasingly positive phase angles over time (Figure 8). Phase angle was more positive in R6/2 than WT mice from 16 weeks of age in the phase-advance group, (16–17 weeks, p<0.05; 17–19 weeks, p<0.01; 19–20 weeks, p<0.001; Figure 8A), and from 15 weeks of age in the phase-delay group (15–17 weeks, p<0.05; 17–20 weeks, p<0.001; Figure 8B). The greater differences between R6/2 and WT mice in the phase-delay group suggest that R6/2 mice adapt better to phase-advance and reversal than phase-delay and reversal. It is interesting to note that the phase angle of entrainment of R6/2 mice during both the phase-delay interval and the reversal from phase-advance (which was, in effect, a phase-delay) appeared to be returning to zero at 16 weeks, before drifting upwards again (Figure 8). These findings suggest once again that the circadian period in these mice was shortening, and there was an impairment in photic re-synchronization.

**Figure 9:**
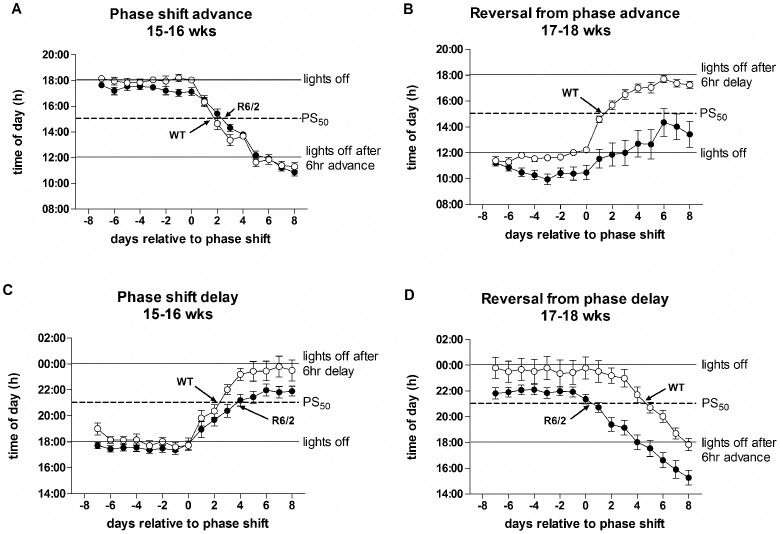
Average time of onset of activity of mice subjected to phase-shift/reversals, showing time to PS_50_ (the time at which half of the phase-shift was achieved). Mice were subjected to either a 6 hour phase-advance (A) followed by a 6 hour reversal (B), or a 6 hour phase-delay (C) again followed by a 6 hour reversal (D). Data are means ± SEM. Open symbols are WT mice, filled symbols are R6/2 mice.

Calculations of PS_50_ suggested that at 15–16 weeks, WT and R6/2 mice adjusted similarly to phase-advance shifts (p>0.05; Figure 9A, Table 3), but on phase-delay shifts, R6/2 mice took longer than WT mice to reach PS_50_ Figure9C, Table 3). On reversal, WT mice adjusted to the phase-delay faster than they did to the phase-advance (p<0.001; Figure 9B & D, Table 3). R6/2 mice, however, showed an impairment on reverting to the normal LD cycle from the phase-advance, and in fact failed to reach PS_50_ (Figure 9B). On reversal from the phase-delay, R6/2 mice reached PS_50_ faster than WT mice (Table 3), although this was due to the time of onset of these mice having already drifted close to the PS_50_ value by day 0. These data suggest again that the circadian period of the R6/2 mice at this age was running at less than 24 hours, and that there was a lack of photic re-synchronization.

**Table 3:** Intra- and inter- genotype comparison of number of days to PS_50_ in the shift/reversal experiment.

	Number of days to PS_50_ (mean ± SEM)		
	WT	R6/2	p value
Phase-advance	2.1±0.3	2.0±0.3	p = 0.85 (n.s.)
Phase-delay	1.2±0.2	2.3±0.4	p<0.05
p value	p = 0.07(n.s.)	p = 0.48 (n.s.)	
Reversal from phase-advance	1.8±0.4	Did not reachPS_50_	n.a.
Reversal from phase-delay	4.5±0.3	1.4±0.3	
p value	p<0.001	n.a.	p<0.001

PS_50_ = number of days for the onset of activity of the mice to advance by 3 hours during each 6 hour phase-shift.

**Figure 10:**
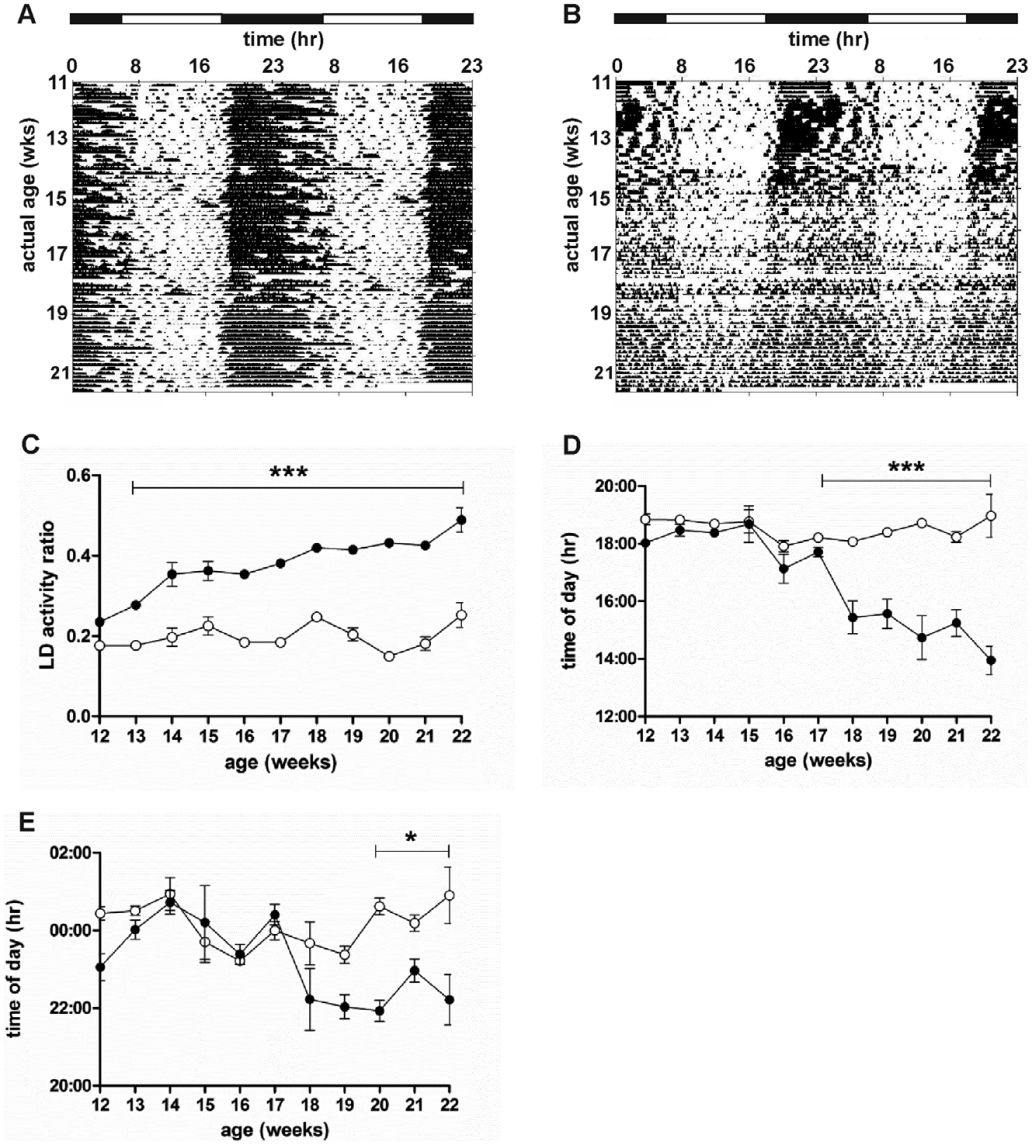
Activity in mice kept under a 23 hour LD cycle. Double-plotted actograms from representative WT (A) and R6/2 (B) mice. Each circadian cycle is 23 hours long, so every line of the double-plotted actograms represents 46 hours. Light/dark activity ratio (C), onset of activity (D), and acrophase (E) were averaged across 7 days. Open symbols are WT mice, filled symbols are R6/2 mice. Data are means ± SEM. * = p<0.05, *** = p<0.001.

We investigated the apparent shortening of the circadian period in R6/2 mice by keeping a cohort under a 23 hour LD cycle. Our data suggested that initially R6/2 mice entrained well to a shorter day (representative actograms are shown in Figure 10A and B). However, as seen under a 24 hour LD regime, the cycle of light/dark activity in R6/2 mice began to break down, as shown by the lack of distinction between periods of rest and activity (Figure 10B). This was confirmed by the increasing LD ratio in R6/2 mice from 13 weeks of real age (p<0.001 relative to WT mice, Figure 10C). Earlier onsets of activity were seen from 17 weeks of real age in R6/2 mice (p<0.001, Figure 10d). Acrophase was also significantly advanced in R6/2 mice from 20 weeks of real age (p<0.05, Figure 10E). These data are similar to those obtained from R6/2 mice held under normal 12∶12 LD conditions (Figure 1), and support our theory that the endogenous period of R6/2 mice continues to shorten with age and phenotype development.

**Figure 11:**
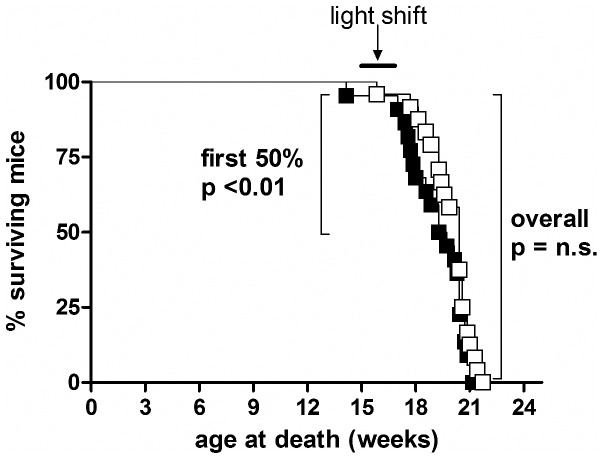
Survival data from R6/2 mice in the shift/reversal experiment. There was no overall effect of experimental condition on survival. However, in the phase-advance group (filled symbols), the first 50% of mice died significantly sooner than those in the phase-delay group (open symbols).

There was no overall difference in age at death between R6/2 mice subjected to phase-advance or phase-delay (Figure 11). However, a comparison of the first 50% of mice to die revealed that R6/2 mice in the phase-advance group died significantly earlier (log-rank test, p<0.01). Survival of WT mice was not measured.

## Discussion

As expected [3], [8]–[9], under normal 12∶12 LD conditions, R6/2 mice start to show a breakdown in their daily cycling from approximately 12–15 weeks of age (shown by the absence of a robust circadian rhythm and the increased ratio of light/dark activity). By 16 weeks of age they showed no clear daily rhythm. However, when the mice were subjected to a series of 4 hour light phase-shifts at 2 week intervals, R6/2 mice adjusted to the new onsets of light and dark, and retained a significant daily rhythmicity at 16 weeks of age. These data suggest not only that R6/2 mice are capable of adapting to changes in the light/dark cycle, but also that the process of doing so may halt the disintegration of daily behavioral rhythmicity. When we challenged the mice with a shift/reversal paradigm, we found that mice of both genotypes adjusted to the phase-advance as quickly as they did to the phase-delay. It was particularly interesting to see that the R6/2 mice clearly entrained to a 6 hour phase-shift at an age where their daily cycling of light/dark activity was typically breaking down (16 weeks). Indeed, when challenged at 17–18 weeks of age by a reversal of 6 hour phase-shifts back to the “normal” LD cycle, the R6/2 mice showed evidence of re-entraining. These results strongly support our hypothesis that although R6/2 mice exhibit disrupted daily rhythms, the underlying molecular machinery is intact and responsive to change.

R6/2 mice took longer to re-entrain following a phase-delay than a phase-advance, in contrast to what we saw with the WT control mice and what has been described by others [18]. This may be explained by dysfunction of the clock genes in the R6/2 mouse. It has been suggested that the clock genes *mPer1* and *mPer2* are critically important in the photic entrainment pathway. These genes regulate expression of *mCry1*, which is thought to gradually reset locomotor behavior following phase-shifts [18]. We have demonstrated dysregulation of the *mPer* genes in the brains of symptomatic R6/2 mice [3], [8]–[9], and a similar dysregulation of *mCry1* in the liver of R6/2 mice [9]. It is possible that the altered expression of *mPer1* and *mPer2* affects expression of *mCry1*. As *mCry1* in normal mice is upregulated following phase-delays, it is possible that the slow re-entrainment to phase-delay seen in R6/2 mice is a consequence of the disrupted cycling of *mPer* genes and a knock-on effect on mCry production and subsequent resetting of locomotor behavior.

An alternative explanation for the slow re-entrainment is that “masking” occurs less readily in aged R6/2 mice than in WT mice. Masking is the phenomenon where bright light causes acute suppression of locomotor activity [19]. If old R6/2 mice are less sensitive to evening light than WT mice, then their activity would not be suppressed to the same degree (partial masking), and the result would be an apparent delay in re-entrainment. It should be noted that even though R6/2 mice exhibited an impairment in re-synchronization, they are still capable of maintaining a daily periodicity, as was seen in our phase-shift experiment using 4 hour phase-advances. Again, this suggests that challenging the mechanisms underlying maintenance of daily rhythmicity in R6/2 mice has beneficial effects on slowing the breakdown in light/dark activity cycles.

We found a significant effect of jet-lag on mortality in R6/2 mice, in that the 6 hour phase-advance caused a significantly earlier age at death in a sub-group of mice (the first 50% to die). It has been reported that jet-lag can cause earlier death in mice, with those subjected to phase-advance being particularly vulnerable [13]. It is not known what caused this increase in mortality, although jet-lag paradigms in rodents have been linked with cardiomyopathy [20], accelerated tumor growth [21] and dysregulation of immune responses [22]. The relevance of these to humans is not clear although shift work, which is analogous to chronic jet-lag, is known to have detrimental effects on health and survival (for review, see [23]).

In the context of HD patients crossing time zones, it may be of concern that while the mice adapted reasonably well to the first light shift, they were impaired in the reversal. If this translates to humans, it is possible that HD patients may be able to adapt to a time-shift involved in flying east, but they would be less able to adjust back on their return journey. However, it should be noted that the phenotype of our mice was already quite advanced when they underwent the reversal at 17 weeks of age; early symptomatic mice, and patients in the early stages of the disease may not have the same problems adjusting to phase shifts. It would be interesting to repeat the experiment with younger mice to see whether this is the case. It would also be useful to know whether HD patients have difficulties adjusting to time zone changes, but to our knowledge there is no documentation concerning jet-lag in HD.

The inability of R6/2 mice to re-entrain robustly following the reversal of a light phase-shift can be largely explained by dysregulated circadian clock genes [3], [8]–[9]. However, we also know from a previous study that 16 week old R6/2 mice kept under DD conditions have a significantly shorter period (23.2 hours) than WT mice (23.8 hours) [17]. However, a change in tau does not fully explain the shortened period under LD conditions. In normal animals, the circadian period is re-synchronized daily by exposure to light [24]–[25]. If an animal is unresponsive to light, the period will not be re-synchronized. To examine this in R6/2 mice, we kept a group of mice under a 23 hour LD cycle. If the period of symptomatic R6/2 mice is fixed at 23 hours [17], we would expect the breakdown in daily activity rhythms to be prevented or, apparently, “rescued” if the mice were kept on a 23 hour day. However, although the R6/2 mice entrained well to a shorter day, their activity rhythms still broke down. Furthermore, onsets and acrophase of activity continued to advance. This suggests that the endogenous period in symptomatic R6/2 mice continues to shorten as they age and their phenotype worsens, and they may also become increasingly light-insensitive. In R6/2 mice, a developing insensitivity to light could result in the failure of the daily photic re-synchronization. It would be interesting to test this by examining the shape of the phase-response curve in R6/2 mice. It is known that R6/2 mice suffer retinal degeneration [10], so it is possible that as the phenotype develops, the mice need a greater photic input to achieve synchronization. Reduced sensitivity to light could also lead to impairment in masking, which in turn may explain the slow re-entrainment to phase-delay seen in R6/2 mice. It would be interesting to examine this, especially as light therapy is currently being tested for its effect on mood and sleep disturbances in Alzheimer’s disease patients [26]. It should be noted that in another mouse model, the BACHD mouse (a mixed CAA-CAG repeat construct), mice at approximately 6 months of age showed delayed re-synchronization to 6 hour phase-advances and delays compared to WT mice [27], although retinal changes have not been reported in these mice.

It has been shown that C57 mice have mutations in both AA-NAT and HIOMT genes, and so cannot produce melatonin [28]. Therefore, we have to consider the possibility that some of the variation in our results can be explained by individual mice being either producers or non-producers of melatonin. For example, the arrhythmicity of aged R6/2 mice may be a consequence of these mice being melatonin non-producers. Since the ratio of melatonin producers/non-producers should be the same in both genotypes, we would then expect to see arrhythmia in young R6/2 mice, and in WT mice. This has not been observed in either this study, or our previous work using R6/2 mice [3], [8]–[9], [23], so we are confident that our observed genotype differences are not due to the background strain. Nevertheless, it would be useful in future to determine whether or not these mice produce melatonin, particularly since drugs targeting the melatonin system are proposed as a therapy for treating circadian dysfunction [29]. It would also be interesting to study the circadian cycling of melatonin production in R6/2 mice, since it has been shown that while HD patients and control subjects have similar diurnal levels of melatonin [30]–[31], the evening rise in melatonin level is delayed in HD patients [31].

In summary, we have shown that although R6/2 mice under normal LD conditions begin to show breakdown of their daily cycle of activity at around 12 weeks, they are still capable of entraining to shifts in light phase up to 16 weeks of age. R6/2 mice showed reduced ability to re-entrain to a light shift reversal at 17 weeks, but still showed clear, if impaired, daily rhythms of activity at 19 weeks. We suggest that in addition to the mechanism that leads ultimately to the complete breakdown of circadian rhythmicity in these mice, there may be a second component involving progressive light insensitivity, which prevents the normal daily re-setting of period length.

## Supporting Information

Figure S1 Additional double-plotted actograms from mice kept under constant LD conditions. Column A are WT mice, column B are R6/2 mice.
(TIF)

Figure S2 Additional double-plotted actograms from WT mice subjected to repeated 4 hour phase-advances (shaded blocks).
(TIF)

Figure S3 Additional double-plotted actograms from representative R6/2 mice subjected to repeated 4 hour phase-advances (shaded blocks).
(TIF)

Figure S4 Additional double-plotted actograms from mice subjected to phase-advance and reversal (shaded blocks). Representative WT mice are shown in (A), and R6/2 mice in (B).
(TIF)

Figure S5 Additional double-plotted actograms from mice subjected to phase-delay and reversal (shaded blocks). Representative WT mice are shown in (A), and R6/2 mice in (B).
(TIF)

Table S1 Time and duration of power cuts during the experiments. Timings are rounded to the nearest 30 minutes.
(DOCX)
