# Supplementary figures and images for: Adaptation to Experimental Jet-Lag in R6/2 Mice despite Circadian Dysrhythmia
Source: PLoS One. 2013 Feb 4;8(2):e55036. doi: 10.1371/journal.pone.0055036 (PMC3563662; doi:10.1371/journal.pone.0055036)

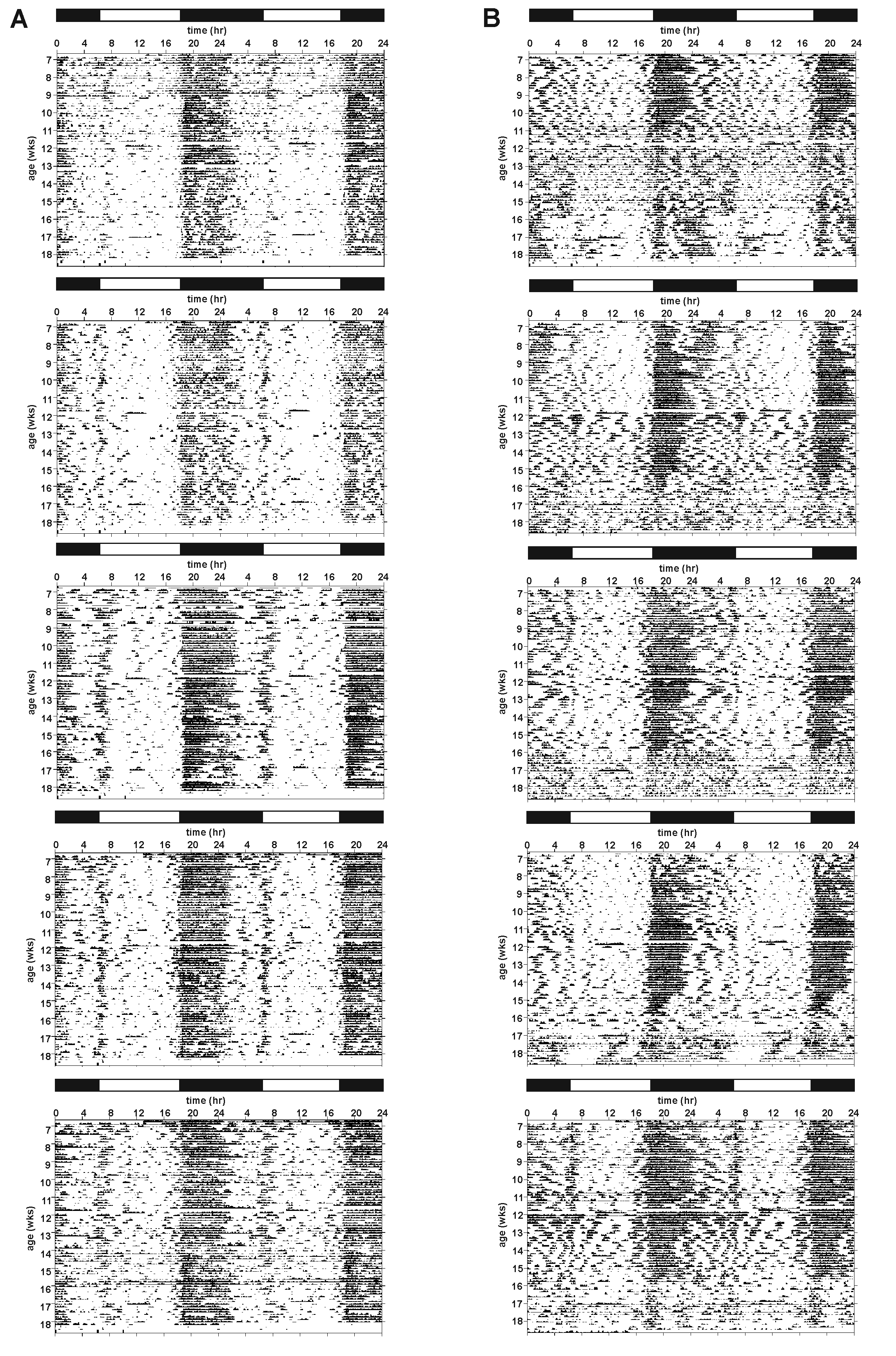

Supplement: Figure S1 — Additional double-plotted actograms from mice kept under constant LD conditions. Column A are WT mice, column B are R6/2 mice. (TIF) [file pone.0055036.s001.tif]

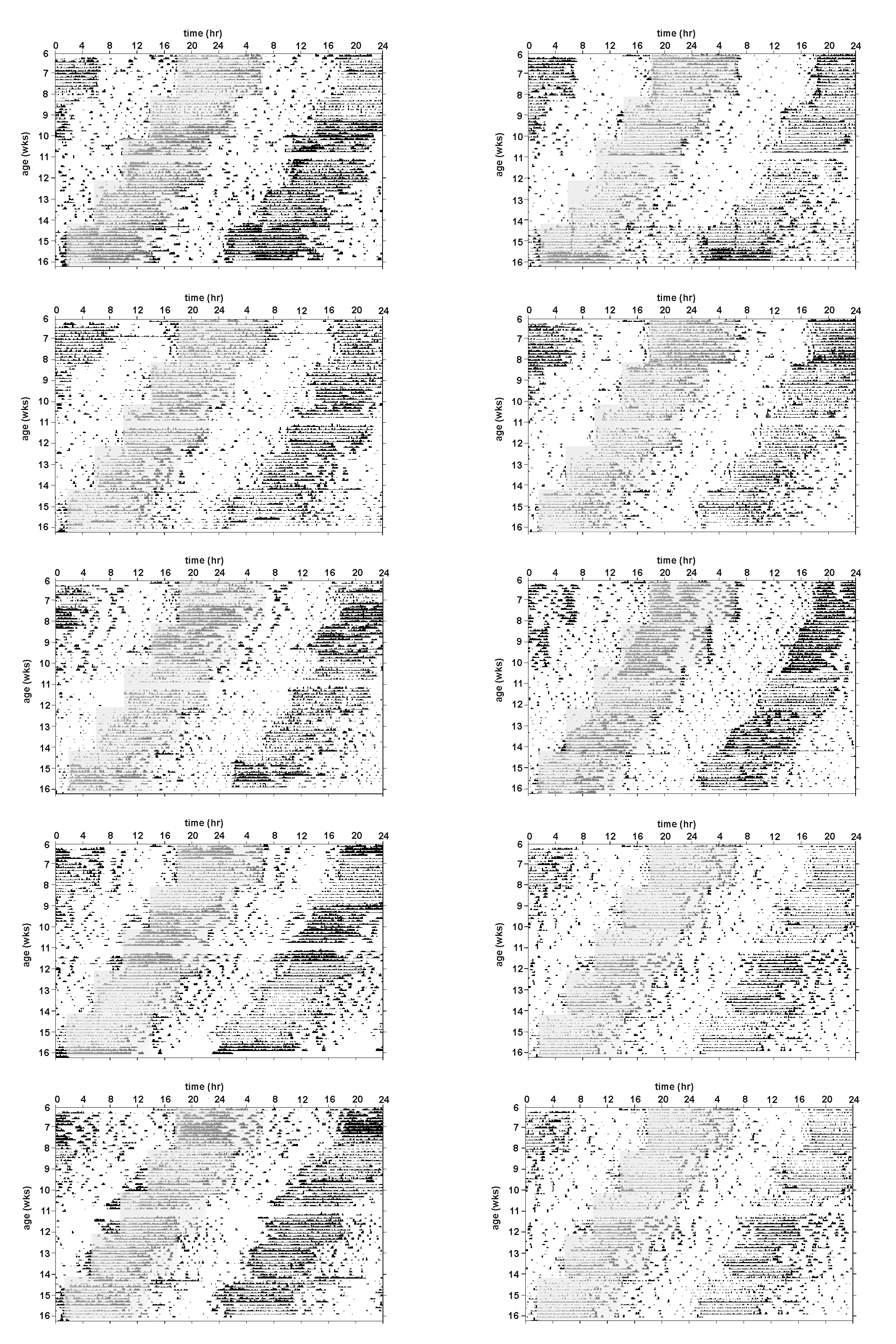

Supplement: Figure S2 — Additional double-plotted actograms from WT mice subjected to repeated 4 hour phase-advances (shaded blocks). (TIF) [file pone.0055036.s002.tif]

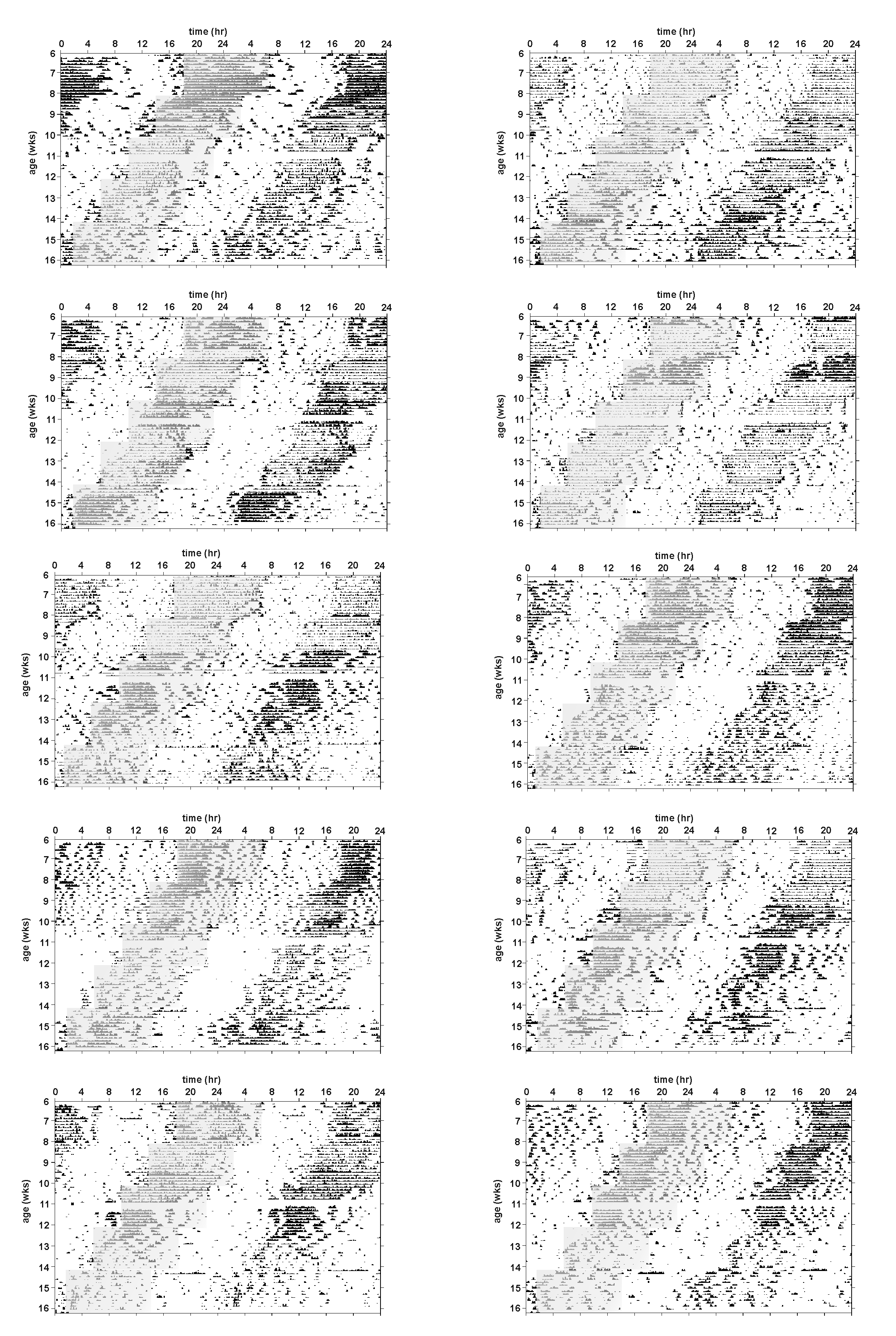

Supplement: Figure S3 — Additional double-plotted actograms from representative R6/2 mice subjected to repeated 4 hour phase-advances (shaded blocks). (TIF) [file pone.0055036.s003.tif]

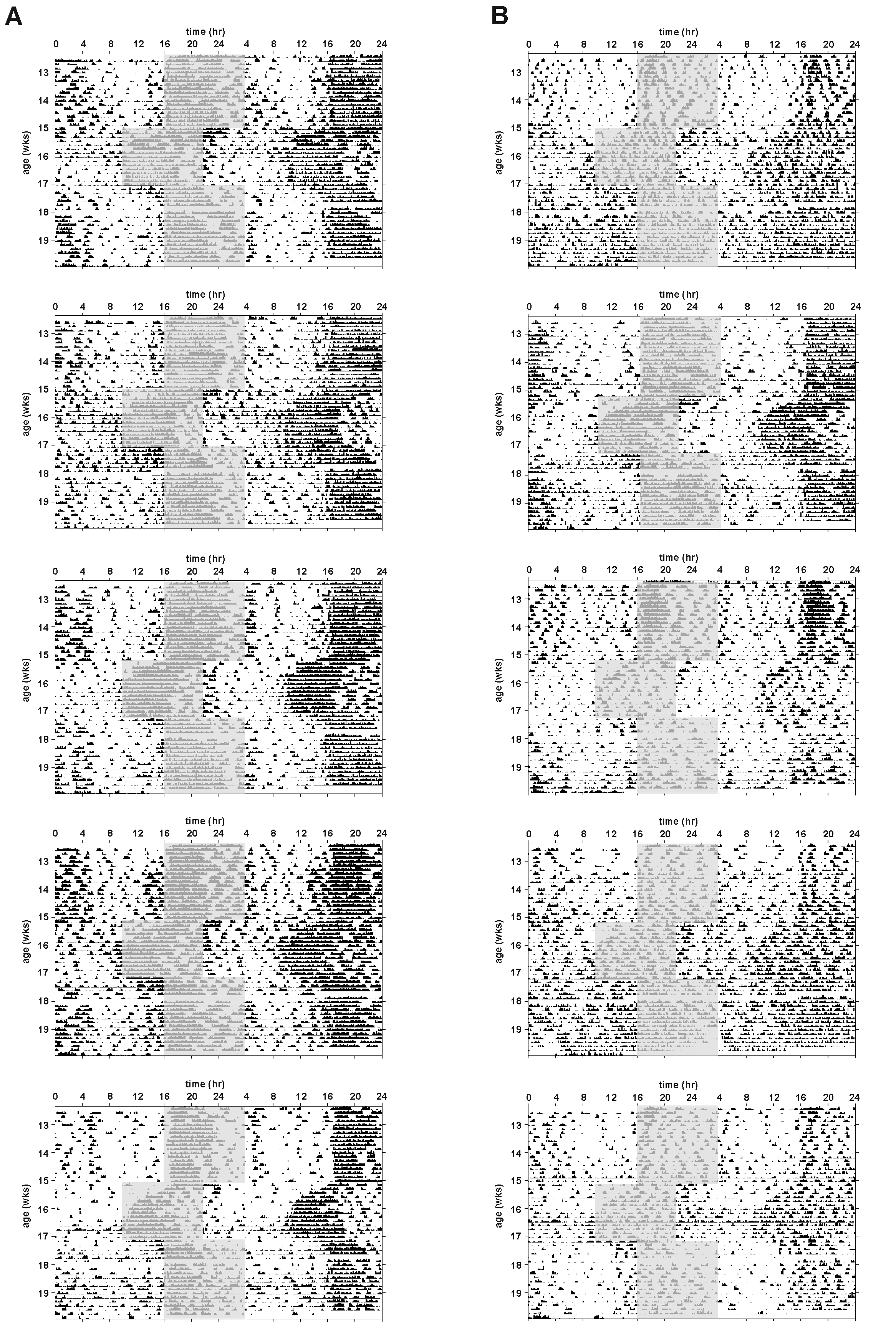

Supplement: Figure S4 — Additional double-plotted actograms from mice subjected to phase-advance and reversal (shaded blocks). Representative WT mice are shown in (A), and R6/2 mice in (B). (TIF) [file pone.0055036.s004.tif]

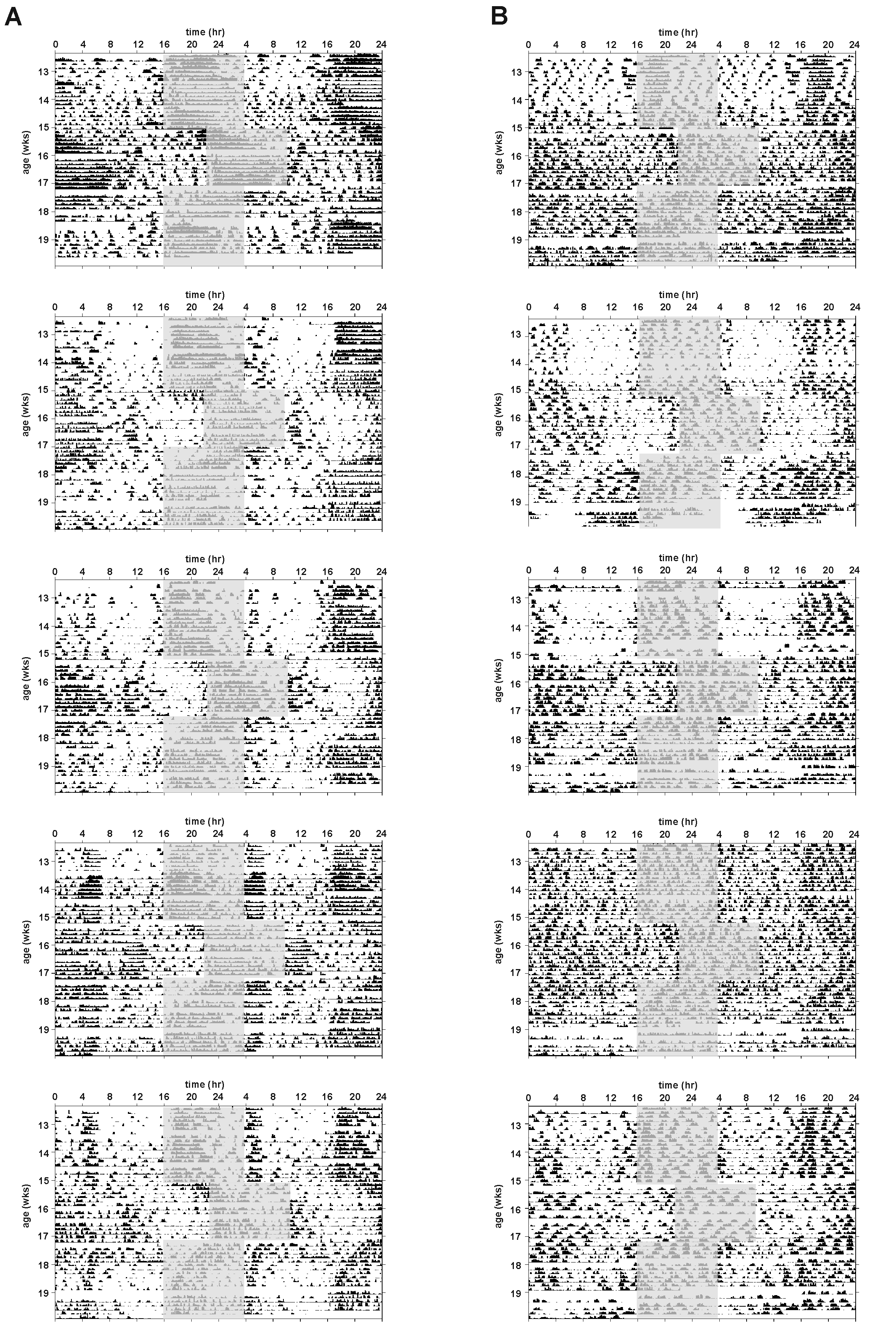

Supplement: Figure S5 — Additional double-plotted actograms from mice subjected to phase-delay and reversal (shaded blocks). Representative WT mice are shown in (A), and R6/2 mice in (B). (TIF) [file pone.0055036.s005.tif]
