# Supplementary material for: Adaptation to Experimental Jet-Lag in R6/2 Mice despite Circadian Dysrhythmia
Source: PLoS One. 2013 Feb 4;8(2):e55036. doi: 10.1371/journal.pone.0055036 (PMC3563662; doi:10.1371/journal.pone.0055036)
Supplement: Table S1 — Time and duration of power cuts during the experiments. Timings are rounded to the nearest 30 minutes. (DOCX) [file pone.0055036.s006.docx]

| **Experiment** | **Average age of mice (weeks.days)** | **Start of power cut** | **End of power cut** | **Duration of power cut (hours)** |
| --- | --- | --- | --- | --- |
| **Normal light/dark** | 12.0 - 12.1 | 16:30 | 08:30 | 16 |
| **Repeated phase advances** | 10.6-11.0 | 06:00 | 10:00 | 28 |
| **Shift/reversal** | 17.6 | 14:00 | 24:00 | 10 |
|  | 18.0 | 02:00 | 15:00 | 15 |
